# Supplementary material for: Early life stress causes sex-specific changes in adult fronto-limbic connectivity that differentially drive learning
Source: eLife. 2020 Dec 1;9:e58301. doi: 10.7554/eLife.58301 (PMC7725504; doi:10.7554/eLife.58301)
Supplement: Source data 1. [file elife-58301-data1.docx]

**Codes written in R for the linear mixed-effects model and output for the different behavioral tests.**

> model <- lmer(min5 ~ (1|litter) + sex + condition)

> summary(model)

Linear mixed model fit by REML. t-tests use Satterthwaite's method ['lmerModLmerTest']

Formula: min5 ~ (1 | litter) + sex + condition

REML criterion at convergence: 591.8

Scaled residuals:

Min 1Q Median 3Q Max

-1.9746 -0.5489 -0.2366 0.4574 2.5951

Random effects:

Groups Name Variance Std.Dev.

litter (Intercept) 61.31 7.83

Residual 237.49 15.41

Number of obs: 72, groups: litter, 11

Fixed effects:

Estimate Std. Error df t value Pr(>|t|)

(Intercept) 27.571 5.075 8.034 5.433 0.000612 ***

sex 6.445 3.904 67.464 1.651 0.103413

condition -17.784 6.221 7.442 -2.859 0.022851 *

---

Signif. codes: 0 ‘***’ 0.001 ‘**’ 0.01 ‘*’ 0.05 ‘.’ 0.1 ‘ ’ 1

Correlation of Fixed Effects:

(Intr) sex

sex -0.291

condition -0.716 -0.105

**NOR**

> model <- lmer(time~ (1|litter) + sex + condition)

> summary(model)

Linear mixed model fit by REML. t-tests use Satterthwaite's method ['lmerModLmerTest']

Formula: time ~ (1 | litter) + sex + condition

REML criterion at convergence: 475.9

Scaled residuals:

Min 1Q Median 3Q Max

-2.2141 -0.6820 -0.0448 0.4603 3.5619

Random effects:

Groups Name Variance Std.Dev.

litter (Intercept) 6.135 2.477

Residual 37.798 6.148

Number of obs: 74, groups: litter, 11

Fixed effects:

Estimate Std. Error df t value Pr(>|t|)

(Intercept) 12.023 1.766 9.125 6.807 7.34e-05 ***

sex 3.871 1.506 70.310 2.571 0.0123 *

condition -4.810 2.134 8.109 -2.254 0.0538 .

---

Signif. codes: 0 ‘***’ 0.001 ‘**’ 0.01 ‘*’ 0.05 ‘.’ 0.1 ‘ ’ 1

Correlation of Fixed Effects:

(Intr) sex

sex -0.366

condition -0.686 -0.085

**L_amy_vHPC**

> model <- lmer(lavhpc ~ (1 | litter) +sex*condition)

> summary(model)

Linear mixed model fit by REML. t-tests use Satterthwaite's method ['lmerModLmerTest']

Formula: lavhpc ~ (1 | litter) + sex * condition

REML criterion at convergence: 344.7

Scaled residuals:

Min 1Q Median 3Q Max

-1.3374 -0.6205 -0.2702 0.2376 1.8065

Random effects:

Groups Name Variance Std.Dev.

litter (Intercept) 211266 459.6

Residual 1119104 1057.9

Number of obs: 24, groups: litter, 8

Fixed effects:

Estimate Std. Error df t value Pr(>|t|)

(Intercept) 10052.500 506.892 9.316 19.832 6.18e-09 ***

sex 2031.814 636.266 19.132 3.193 0.004755 **

condition 1784.728 699.164 11.708 2.553 0.025757 *

sex:condition -3851.208 901.539 19.360 -4.272 0.000397 ***

---

Signif. codes: 0 ‘***’ 0.001 ‘**’ 0.01 ‘*’ 0.05 ‘.’ 0.1 ‘ ’ 1

Correlation of Fixed Effects:

(Intr) sex condtn

sex -0.578

condition -0.725 0.419

sex:condition 0.408 -0.706 -0.597
