# Supplementary material for: Early life stress causes sex-specific changes in adult fronto-limbic connectivity that differentially drive learning
Source: eLife. 2020 Dec 1;9:e58301. doi: 10.7554/eLife.58301 (PMC7725504; doi:10.7554/eLife.58301)
Supplement: Supplementary file 1. — CA – closed arms, EPM – elevated plus maze, OA – open arms, OF – open field test, s – seconds. Significant outcomes are shown in red font. [file elife-58301-supp1.docx]

**Supplementary File 1**. Summary of Behavioral result in Cohort 2. CA- closed arms, EPM- elevated plus maze, OA- open arms, OF- open field test, s- seconds. Significant outcomes are shown in red font.

| **Behavior** | **CTL M Mean**  **(SEM)** | **CTL F**  **Mean**  **(SEM)** | **UPS M**  **Mean**  **(SEM)** | **UPS F**  **Mean**  **(SEM)** | **Main Effect**  **of Rearing** | **Main Effect**  **of Sex** | **Sex X Rearing**  **Interaction** |
| --- | --- | --- | --- | --- | --- | --- | --- |
| Time in Center (OF) (s) | 23.39 (1.86)  n = 18 | 21.46 (1.88)  n = 16 | 19.56 (3.29)  n = 9 | 22.06 (2.21)  n = 19 | F (1, 58) = 0.46, *p* = 0.50 | F (1, 58) = 0.01, *p* = 0.90 | F (1, 58) = 0.86, *p* = 0.36 |
| Distance Traveled (OF) (cm) | 3450.82 (71.65)  n = 18 | 3760.85 (443.22)  n = 16 | 3376.99 (200.95)  n = 9 | 3159.42 (118.47)  n = 19 | F (1, 58) = 1.56, *p* = 0.22 | F (1, 58) = 0.03, *p* = 0.87 | F (1, 58) = 0.95, *p* = 0.87 |
| OA Duration (EPM) (s) | 96.54 (15.17)  n = 19 | 80.69 (13.15) n = 17 | 135.71 (21.41)  n = 9 | 97.26 (18.30)  n = 20 | F (1, 61) = 2.50, *p* = 0.12 | F (1, 61) = 2.37, *p* = 0.13 | F (1, 61) = 0.41, *p* = 0.52 |
| CA Duration (EPM) (s) | 92.04 (11.65)  n = 19 | 115.40 (16.74) n = 17 | 87.76 (17.61)  n = 9 | 95.37 (13.84)  n = 20 | F (1, 61) = 0.63, *p* = 0.43 | F (1, 61) = 1.01, *p* = 0.32 | F (1, 61) = 0.26, *p* = 0.61 |
| Object Exploration (s) | 10.59 (1.12)  n = 16 | 10.07 (1.53) n = 16 | 6.91 (1.76)  n = 8 | 7.66 (1.08)  n = 16 | F (1, 52) = 4.34, *p* = 0.04 | F (1, 52) = 0.01, *p* = 0.94 | F (1, 52) = 0.19, *p* = 0.67 |
| Context Test (% Freezing) | 19.92 (3.39)  n = 17 | 26.29 (4.48) n = 17 | 15.34 (2.96)  n = 12 | 15.89 (3.31)  n = 20 | F (1, 62) = 3.99, *p* = 0.05 | F (1, 62) = 0.86, *p* = 0.36 | F (1, 62) = 0.6, *p* = 0.44 |
| Cued Test (% Freezing) | 26.72 (5.03)  n = 17 | 41.64 (4.99) n = 17 | 32.12 (6.77)  n = 12 | 40.35 (5.84)  n = 20 | F (1, 62) = 0.13, *p* = 0.72 | F (1, 62) = 4.05, *p* = 0.05 | F (1, 62) = 0.34, *p* = 0.56 |
