# Supplementary material for: Early life stress causes sex-specific changes in adult fronto-limbic connectivity that differentially drive learning
Source: eLife. 2020 Dec 1;9:e58301. doi: 10.7554/eLife.58301 (PMC7725504; doi:10.7554/eLife.58301)
Supplement: Supplementary file 2. — To examine whether the reported effects were attributable to litter effect, we conducted linear mixed-effects model using R version 3.6.0 (R Development Core Team, 2019) and the following packages: lme4 version 1.1–21 (Bates et al., 2015) and lmerTest version 3.1–0 (Kuznetsova et al., 2017), with litter included in each model as a random effect. Importantly, these models provided a second test of key behavioral endpoints while controlling for the hierarchical structure of the data as animals were nested within litters. Results again revealed a main effect of rearing condition on contextual fear conditioning with reduced freezing found in UPS- compared to CTL-reared animals, b = −17.78, SE = 6.22, t = −2.86, p=0.02. Similarly, time spent exploring novel objects was reduced in UPS compared to CTL animals, b = −4.81, SE = 2.13, t = −2.25, p=0.053. There was also a main effect of sex in NOR, with females demonstrating greater exploration than males, b = 3.87, SE = 1.51, t = 2.57, p=0.01. The results of the tractography linear mixed-effects model were also consistent with the reported ANOVA, revealing a significant interaction between sex and rearing condition on streamlines between the amygdala and ventral hippocampus in the left hemisphere, b = −3851.21, SE = 901.54, t = −4.27, p=0.0004. Together, the results of these follow-up models support those reported in the main text and suggest that litter (or maternal behavior) had minimal effect on the key dependent measures. R codes and output for the linear mixed model analysis are available in Source data 1. [file elife-58301-supp2.docx]

**Supplementary File 2.** Linear mixed-effect model (LMEM) for key outcomes in UPS mice. To examine whether the reported effects were attributable to *litter effect*, we conducted linear mixed-effects model using R version 3.6.0 (R Core Team, 2019) and the following packages: lme4 version 1.1-21 (Bates et al., 2015) and lmerTest version 3.1-0 (Kuznetsova et al., 2017), with litter included in each model as a random effect. Importantly, these models provided a second test of key behavioral endpoints while controlling for the hierarchical structure of the data as animals were nested within litters. Results again revealed a main effect of rearing condition on contextual fear conditioning with reduced freezing found in UPS- compared to CTL-reared animals, b = -17.78, SE = 6.22, t = -2.86, *p* = 0.02. Similarly, time spent exploring novel objects was reduced in UPS compared to CTL animals, b = -4.81, SE = 2.13, t = -2.25, *p* = 0.053. There was also a main effect of sex in NOR, with females demonstrating greater exploration than males, b = 3.87, SE = 1.51, t = 2.57, *p* = 0.01. The results of the tractography linear mixed-effects model were also consistent with the reported ANOVA, revealing a significant interaction between sex and rearing condition on streamlines between the amygdala and ventral hippocampus in the left hemisphere, b = -3851.21, SE = 901.54, t = -4.27, *p* = 0.0004. Together, the results of these follow-up models support those reported in the main text and suggest that litter (or maternal behavior) had minimal effect on the key dependent measures. R codes and output for the linear mixed model analysis are available in **Source Data 1.**

**References**

BATES, D., MÄCHLER, M., BOLKER, B. & WALKER, S. (2015). Fitting Linear Mixed-Effects Models Using lme4. *Journal of statistical software,* 67.

KUZNETSOVA, A., BROCKHOFF P.B. & CHRISTENSEN, R. H. B. (2017). lmerTest Package: Tests in Linear Mixed Effects Models. *Journal of statistical software,* 82.
