# Supplementary material for: Early life stress causes sex-specific changes in adult fronto-limbic connectivity that differentially drive learning
Source: eLife. 2020 Dec 1;9:e58301. doi: 10.7554/eLife.58301 (PMC7725504; doi:10.7554/eLife.58301)
Supplement: Supplementary file 3. — n = 12 mice per condition. [file elife-58301-supp3.docx]

**Supplementary File 3**. Summary of local volumetric changes induced by UPS using voxel based morphometric analysis with minimal cluster size > 25 voxels, FDR < 0.1, *p* < 0.0105. n = 12 mice per condition.

| **Brain region** | **Direction Compared to CTL** | **Outcomes in humans** | **References for human literature** |
| --- | --- | --- | --- |
| Olfactory Bulb | Increased | Decreased in those that develop PTSD, but only one report | (Nwulia et al., 2017) |
| Nucleus accumbens | Increased | Inconsistent findings | (Teicher and Samson, 2016, Frodl et al., 2017) |
| Cingulate Cortex | Increased | Mostly reduced, but see Zuo 2019 for increased vol. | (Teicher and Samson, 2016, Zuo et al., 2019) |
| Sensory Cortex | Increased | Changes appear to be related to the type of adversity | (Teicher and Samson, 2016) |
| Ventral Hippocampus | Increased | Decreased, but see Frodl 2017 for a failure to replicate in a large cohort. | (Teicher and Samson, 2016, Frodl et al., 2017) |
| Amygdala | Increased | Inconsistent findings | (Teicher and Samson, 2016, VanTieghem and Tottenham, 2018, Frodl et al., 2017) |
| Frontal Cortex | Decreased | Decreased | (Teicher and Samson, 2016) |
| Fimbria | Decreased | Inconsistent findings | (Teicher et al., 2012, Frodl et al., 2017) |
| Striatum | Decreased | Inconsistent findings | (Teicher and Samson, 2016, Frodl et al., 2017) |
| Thalamus | Decreased | Inconsistent findings | (Teicher and Samson, 2016, Frodl et al., 2017) |

**References**

FRODL, T., JANOWITZ, D., SCHMAAL, L., TOZZI, L., DOBROWOLNY, H., STEIN, D. J., VELTMAN, D. J., WITTFELD, K., VAN ERP, T. G. M., JAHANSHAD, N., BLOCK, A., HEGENSCHEID, K., VOLZKE, H., LAGOPOULOS, J., HATTON, S. N., HICKIE, I. B., FREY, E. M., CARBALLEDO, A., BROOKS, S. J., VULETIC, D., UHLMANN, A., VEER, I. M., WALTER, H., SCHNELL, K., GROTEGERD, D., AROLT, V., KUGEL, H., SCHRAMM, E., KONRAD, C., ZUROWSKI, B., BAUNE, B. T., VAN DER WEE, N. J. A., VAN TOL, M. J., PENNINX, B., THOMPSON, P. M., HIBAR, D. P., DANNLOWSKI, U. & GRABE, H. J. (2017). Childhood adversity impacts on brain subcortical structures relevant to depression. *J Psychiatr Res,* 86**,** 58-65.

NWULIA, E. A., RAI, N., SARTIP, K., HIPOLITO, M. M. S., MCLEAN, C. K., FLANAGAN, K., HAMILTON, F., LAMBERT, S., LE, H. N., VANMETER, J. & KAPETANOVIC, S. (2017). A Pilot Study of Reduced Olfactory Bulb Volume as a Marker of PTSD in Childhood Trauma-Exposed Adult HIV-Infected Patients. *J Trauma Stress,* 30**,** 537-544.

TEICHER, M. H., ANDERSON, C. M. & POLCARI, A. (2012). Childhood maltreatment is associated with reduced volume in the hippocampal subfields CA3, dentate gyrus, and subiculum. *Proc Natl Acad Sci U S A,* 109**,** E563-572.

TEICHER, M. H. & SAMSON, J. A. (2016). Annual Research Review: Enduring neurobiological effects of childhood abuse and neglect. *J Child Psychol Psychiatry,* 57**,** 241-266.

VANTIEGHEM, M. R. & TOTTENHAM, N. (2018). Neurobiological Programming of Early Life Stress: Functional Development of Amygdala-Prefrontal Circuitry and Vulnerability for Stress-Related Psychopathology. *Curr Top Behav Neurosci,* 38**,** 117-136.

ZUO, P., WANG, Y., LIU, J., HU, S., ZHAO, G., HUANG, L. & LIN, D. (2019). Effects of early adversity on the brain: Larger-volume anterior cingulate cortex in AIDS orphans. *PLoS ONE,* 14**,** e0210489.
