# Supplementary material for: Early life stress causes sex-specific changes in adult fronto-limbic connectivity that differentially drive learning
Source: eLife. 2020 Dec 1;9:e58301. doi: 10.7554/eLife.58301 (PMC7725504; doi:10.7554/eLife.58301)
Supplement: Transparent reporting form [file elife-58301-transrepform.docx]

***eLife’s* transparent reporting form**

We encourage authors to provide detailed information *within their submission* to facilitate the interpretation and replication of experiments. Authors can upload supporting documentation to indicate the use of appropriate reporting guidelines for health-related research (see [EQUATOR Network](http://www.equator-network.org/%20)), life science research (see the [BioSharing Information Resource](https://biosharing.org/)), or the [ARRIVE guidelines](http://www.plosbiology.org/article/info:doi/10.1371/journal.pbio.1000412) for reporting work involving animal research. Where applicable, authors should refer to any relevant reporting standards documents in this form.

If you have any questions, please consult our Journal Policies and/or contact us: [editorial@elifesciences.org](mailto:editorial@elifesciences.org).

**Sample-size estimation**

- You should state whether an appropriate sample size was computed when the study was being designed
- You should state the statistical method of sample size computation and any required assumptions
- If no explicit power analysis was used, you should describe how you decided what sample (replicate) size (number) to use

Please outline where this information can be found within the submission (e.g., sections or figure legends), or explain why this information doesn’t apply to your submission:

Assumptions for a priori power calculations are outlined in the method section (lines 733-737, and 800-801) and were conducted using G*Power.

**Replicates**

- You should report how often each experiment was performed
- You should include a definition of biological versus technical replication
- The data obtained should be provided and sufficient information should be provided to indicate the number of independent biological and/or technical replicates
- If you encountered any outliers, you should describe how these were handled
- Criteria for exclusion/inclusion of data should be clearly stated
- High-throughput sequence data should be uploaded before submission, with a private link for reviewers provided (these are available from both GEO and ArrayExpress)

Please outline where this information can be found within the submission (e.g., sections or figure legends), or explain why this information doesn’t apply to your submission:

The behavioral work was conducted using a large cohort of adult mice (n= 12-23 mice per rearing condition and sex, from 4-6 independent litters) and was replicated using a 2^nd^ cohort of mice (n= 9-19 mice per group, from 4-6 independent litters) and a 3^rd^ cohort of mice (n= 13-15 per group, from 4-6 litters). This setup is described in the method section (lines 737-742), the results section (lines 183-243), Figure 2 Supplementary Figure 2, and Supplementary File 1.

The structural connectivity data in our initial cohort (Fig 5-6, n=6 mice per group) is a replication of our previous work using resting state fMRI (Johnson 2018) and further confirmed these findings using a larger cohort of mice (n-12-13 mice per group, Figs 10-12).

Only biological samples were used (no technical replicates were included). This issue is not mentioned in the text because it is not relevant to this paper.

Animals that were > 2 s.d. above or below the mean were eliminated from behavioral analysis (see Statistical analysis lines 867-868).

**Statistical reporting**

- Statistical analysis methods should be described and justified
- Raw data should be presented in figures whenever informative to do so (typically when N per group is less than 10)
- For each experiment, you should identify the statistical tests used, exact values of N, definitions of center, methods of multiple test correction, and dispersion and precision measures (e.g., mean, median, SD, SEM, confidence intervals; and, for the major substantive results, a measure of effect size (e.g., Pearson's r, Cohen's d)
- Report exact p-values wherever possible alongside the summary statistics and 95% confidence intervals. These should be reported for all key questions and not only when the p-value is less than 0.05.

Please outline where this information can be found within the submission (e.g., sections or figure legends), or explain why this information doesn’t apply to your submission:

Statistical analyses are described in Method section (lines 864- 885) and throughout the results section. The results sections include all the necessary information including sample size, tests used, and effect size. 95% confidence intervals are shown for the moderator mediation analysis. To improve readability and to shorten the manuscript, 95% confidence intervals are not shown for other sections of the results. Means and SEM are shown in all figures and are explicitly mentioned in the captions. Raw data is shown in all figures with sample sizes of n< 10 (Figs 1 E-F, 5 A-B, 6 A-C, 8 B-C, 9 B-D) and supplemental figures (Figure 5 Supplementary Figures 2-3, Figure 6 Supplementary Figure 1).

The cluster sizes and false discovery rates used for the volumetric voxel analysis are mentioned in the results sections lines (249, 262, 267), Figure 3, and Figure 3 Supplementary Figs 1-2. The cluster sizes and false discovery rates used for the fractional anisotropy analysis are mentioned in the results sections lines (277) and legends of Figs 4, Fig 4 supplementary figs 1-2)

(For large datasets, or papers with a very large number of statistical tests, you may upload a single table file with tests, Ns, etc., with reference to sections in the manuscript.)

**Group allocation**

- Indicate how samples were allocated into experimental groups (in the case of clinical studies, please specify allocation to treatment method); if randomization was used, please also state if restricted randomization was applied
- Indicate if masking was used during group allocation, data collection and/or data analysis

Please outline where this information can be found within the submission (e.g., sections or figure legends), or explain why this information doesn’t apply to your submission:

Litters were randomized to either UPS or control conditions (Method section, lines 716-717). No restricted randomization was used.

Behavioral outcomes for the open field, elevated plus maze, and fear conditioning were measured using automated scoring obviating concerns for biased scoring (lines 752-759). Scoring for the novel object exploration was done by an experimenter blind to the sex and rearing condition (lines 762-764).

Images were acquired and processed by an experimenter blind to the sex and rearing condition (Line 807-808)

**Additional data files (“source data”)**

- We encourage you to upload relevant additional data files, such as numerical data that are represented as a graph in a figure, or as a summary table
- Where provided, these should be in the most useful format, and they can be uploaded as “Source data” files linked to a main figure or table
- Include model definition files including the full list of parameters used
- Include code used for data analysis (e.g., R, MatLab)
- Avoid stating that data files are “available upon request”

Please indicate the figures or tables for which source data files have been provided:

The following source data are available:

- **Figure 1-source data.** Raw data for body weight, corticosterone level, and adrenal weight.
- **Figure 2-source data**. Raw data for behavioral outcomes.
- **Source Data 1.** R codes and output for the linear mixed model analysis.
- **Figure 3-source data.** Matlab codes used to conduct the 2 X 2 analyses for figures 3 and 4.
- **Figure 7-source data**. SPSS syntax used for the moderator analysis.
- **Figure 8-source data**. GRETNA codes used for global connectivity analysis.
- **Figure 9-source data**. GRETNA codes used to assess amygdala connectivity.
- **Figure 10-source data**. Raw data for fronto-limbic connectivity in the extended cohort.
- **Figure 11-source data**. Raw data for figures 11 and 12.
